# Left ventricular unloading to facilitate ventricular remodelling in heart failure: A narrative review of mechanical circulatory support

**DOI:** 10.1113/EP091796

**Published:** 2024-10-14

**Authors:** Fatima Kayali, Owais Tahhan, Guglielmo Vecchio, Matti Jubouri, Judi M. Noubani, Damian M. Bailey, Ian M. Williams, Wael I. Awad, Mohamad Bashir

**Affiliations:** ^1^ University Hospitals Sussex NHS Foundation Trust Brighton and Hove Sussex UK; ^2^ Aston Medical School Aston University Birmingham UK; ^3^ Hull York Medical School University of York York UK; ^4^ Faculty of Medicine Jordan University of Science and Technology Irbid Jordan; ^5^ Neurovascular Research Laboratory, Faculty of Life Sciences and Education University of South Wales Pontypridd UK; ^6^ Department of Vascular Surgery University Hospital of Wales Cardiff UK; ^7^ Department of Cardiothoracic Surgery, Barts Heart Centre St Bartholomew's Hospital London UK

**Keywords:** heart failure, left ventricular unloading, mechanical circulatory support, ventricular remodelling

## Abstract

Heart failure represents a dynamic clinical challenge with the continuous rise of a multi‐morbid and ageing population. Yet, the evolving nature of mechanical circulatory support offers a variety of means to manage candidates who might benefit from such interventions. This narrative review focuses on the role of the main mechanical circulatory support devices, such as ventricular assist device, extracorporeal membrane oxygenation, Impella and TandemHeart, in the physiological process of ventricular unloading and remodelling in heart failure, highlighting their characteristics, mechanism and clinical outcomes. The outcome measures described include physiological changes (i.e., stroke volume or preload and afterload), intracardiac pressure (i.e., end‐diastolic pressure) and extracardiac pressure (i.e., pulmonary capillary wedge pressure). Overall, all the above mechanical circulatory support strategies can facilitate the unloading of the ventricular failure through different mechanisms, which subsequently affects the ventricular remodelling process. These physiological changes start immediately after ventricular assist device implantation. The devices are indicated in different but overlapping populations and operate in distinctive ways; yet, they have evidenced performance to a favourable standard to improve cardiac function in heart failure, although this proved variable for different devices, and further high‐quality trials are vital to assess their clinical outcomes further. Both Impella and TandemHeart are indicated mainly in cardiogenic shock and high‐risk percutaneous coronary intervention patients; at the time the literature was evaluated, both devices were found to yield a significant improvement in haemodynamics but not in survival. Nevertheless, the choice of device strategy should be based on individual patient factors, including indication, to optimize clinical outcomes.

## Abstract (highlights)

- **What is the topic of this review?**
  This narrative review focuses on the role of the main mechanical circulatory support devices in the physiological process of ventricular unloading and remodelling in heart failure.
- **What advances does it highlight?**
  This narrative review presents a comprehensive overview of the main mechanical circulatory support devices in heart failure, highlighting their characteristics, mechanisms and clinical outcomes. These devices can facilitate the unloading of ventricular failure to varying extents and through different mechanisms, which subsequently affects the ventricular remodelling process. Nevertheless, the choice of device strategy should be based on individual patient factors, including indication, to optimize clinical outcomes.

## 1 INTRODUCTION

With the continuous rise of an ageing population, heart failure (HF) continues to affect an estimated 64.3 million people today and has been recognized in the literature as an emerging epidemic for more than two decades (Groenewegen et al., 2020). Heart failure is the inability to maintain an adequate cardiac output to sustain metabolic requirements, as a result of the reduced mean arterial pressure (MAP) and tissue perfusion (National Heart, Lung & Blood Institute, 2022). As a clinical syndrome, it can occur secondary to a variety of chronic diseases, including hypertension, ischaemic heart disease and diabetes. Rarer aetiologies include cardiotoxic drugs, systemic toxins and valvular disease (Kemp & Conte, 2012).

The pathophysiology of this disease occurs owing to injury to the myocardium itself, triggering compensatory mechanisms to overcome the inadequate systemic perfusion. Preload is defined as all elements that impact passive ventricular wall tension at the end of diastole, whilst afterload is all elements that contribute to total myocardial wall tension during systolic ejection (Norton, 2001). True left ventricular (LV) preload has been described as the difference between LV filling pressure and right atrial pressure, measured using an invasive flow‐directed catheter (Tyberg et al., 1986). The main compensatory mechanisms include neurohormonal activation and alteration in autonomic control. Both mechanisms support the heart in normal physiological circumstances; however, they also play a pivotal role in the exacerbation and progression of chronic HF. Activation of the renin–angiotensin–aldosterone system and the sympathetic nervous system is associated with apoptosis of cardiac myocytes, hypertrophy and myocardial necrosis (Jackson et al., 2000). The subsequent volume overload results in maladaptive remodelling, which involves the reorganization of myocytes, vessels and interstitial cells. This results in increased stiffness and impaired contractility (Heusch et al., 2014). With the limit of cardiac transplant accessibility and the reduction in the donor pool, the question of how to manage the growing population with advanced HF resistant to optimal medical treatment is posed (Deng et al., 2001). Other than pharmacological agents, treatment options for HF include percutaneous interventions and surgical procedures to facilitate mechanical circulatory support (MCS) and assist in unloading the strained myocardium. The main focus of this review is on the percutaneous and surgical techniques facilitating ventricular remodelling. Examples of percutaneous techniques include ventriculoarterial extracorporeal membrane oxygenation (VA‐ECMO) and specific versions of the Impella device family, TandemHeart and protekduo. Surgical options include the remainder of the Impella device family and ventricular assist devices (VADs).

This narrative review investigates the aforementioned ventricular unloading techniques in HF, highlighting their characteristics, mechanisms and clinical outcomes. Some of the outcome measures described include physiological changes (i.e., stroke volume or preload and afterload), intracardiac pressure (i.e., end‐diastolic pressure) and extracardiac pressure [i.e., pulmonary capillary wedge pressure (PCWP)] where described in the literature for each device.

## 2 VENTRICULAR ASSIST DEVICES

Ventricular assist devices have undergone significant evolution in the last decade as advancements in available commercial devices have taken place. The role of VADs was established in the 1960s when DeBakey first implanted this technology to manage cardiogenic shock (CS) in a patient undergoing valvular surgery (Sayer et al., 2009). Ventricular assist devices are now indicated as a bridge to therapy or heart transplant or as a destination therapy in cases where the former two options are not suitable. The initial models of VADs were associated with significant risks of complications, including bleeding, infection and haemolysis, and their large size often made the mobilization of patients difficult (Sayer et al., 2009). As the mismatch between the pool of suitable donors and the ageing population grows, ventricular failure will become a more prominent and challenging issue for the interdisciplinary team, and further understanding of ventricular unloading with new devices is paramount.

### 2.1 Left ventricular assist devices

Left VADs (LVADs) offer a diversion of blood from the left atrium or ventricle to the aorta and have been associated with 1‐year survival rates reaching 94% postimplantation and 78% after 3 years (Gerhard et al., 2021; Lim et al., 2017). First‐generation LVADs, such as Thoratec Heartmate I and Berlin Heart's EXCOR, operated using volume‐displacement mechanics (Lund et al., 2010). This was superseded by a continuous flow LVAD, formed of an inflow cannula in the LV, an impeller and the outflow cannula in the aorta (McCarthy et al., 1995). The impeller then accelerates the speed of blood through centrifugal pumps, where the rotating impeller creates a vacuum, such as Heartware VAD and Heartmate III. These machines tend to be smaller, more easily implanted and with fewer parts, including valves and air vents, allowing for more long‐term benefits and reduced complications, including infection and venous thromboembolism (Lund et al., 2010). Additionally, axial flow devices, such as the Heartmate II device, have been introduced to the market, whereby blood moves by positive displacement. Further advancements in these third‐generation devices are exhibited in the availability of mechanical, magnetic or hydro‐operated motors, improving these outcomes further, including decreased incidence of pump thrombosis and reoperation for pump malfunction (Mehra et al., 2017). Table 1 summarizes the differences between some of these devices (Mehra et al., 2017; Patel et al., 2010).

**TABLE 1:** Examples of intracorporeal left ventricular assist devices, their placement, flow type and maximum flow (in litres per minute) (Patel et al., 2010).

Device	Placement	Flow type	Maximum flow (L/min)
Heartmate II	Intracorporeal (surgically placed)	Axial	10
Heartmate XVE		Pulsatile	
Heartware LVAS		Centrifugal	
Jarvik 2000 Flowmaker		Axial	7
Durraheart LVAS		Centrifugal	8

The physiological changes following LVAD implantation are almost immediate. These changes include reductions in the LV filling pressure, pulmonary artery wedge pressure and LV end‐diastolic diameter (Jung et al., 2015; McCarthy et al., 1995). Additionally, as positive remodelling of the myocardium occurs, myocyte size reduces, collagen levels increase, and improved molecular transportation takes place at the level of the sarcoplasmic reticulum during cardiac diastole (Madigan et al., 2001). Post‐LVAD implantation, normalizing haemodynamic pressures, alongside improved molecular remodelling and myocardial contractility, promotes unloading of the failing LV (Burkhoff et al., 2000). Although LVADs are designed to accommodate changes in the preload of the heart, this sensitivity is thought to be only one‐third of the normal human heart (Küçüker et al., 2004). Further in vitro studies are needed to establish the impact of this limitation further.

Küçüker et al. explored the impact of LVAD implantation on the anatomical and physiological changes and alterations in the levels of neurohormones (Kucuker et al., 2004). The reduction in left atrial pressure caused by LVAD placement results in lower right ventricle (RV) afterload and increased RV venous return and, in turn, optimized overall cardiac output (Farrar, 2000; Kret & Arora, 2007; Küçüker et al., 2004). Additionally, it is well established that LVAD implantation reduces catecholamine and pro‐inflammatory cytokine levels, limiting further hypertrophy of the myocardium and fibrosis. In their study, Küçüker et al. (2004) compared LV and RV samples in patients pre‐implantation, intra‐operatively and at the time of LVAD removal. Five patients received a continuous flow device known as the DeBakey Axial Flow VAD, and the remaining five received Novacor's pulsatile flow left ventricular assist system (LVAS). In this study, both RV and LV failure was shown to cause increased production of collagen and myocardial tumour necrosis factor‐α levels, and LVAD support showed reversal and normalization of these levels in both continuous and pulsatile flow devices. Firstly, myocyte size in LV failure was significantly decreased with both LVADs, in contrast to the control group. The difference in myocyte size between the Novocor LVAS in comparison to the no support group was 23.1 ± 1.3 versus 32.3 ± 1.8 µm (*P *< 0.01), and the DeBakey Axial Flow VAD in contrast to the no support group was 21.5 ± 1.5 versus 32.3 ± 1.8 µm (*P *< 0.001), respectively. In contrast, there was no significant difference in collagen levels in the RV in patients with Novocor LVAS, in comparison to patients with no ventricular support (27.6% ± 2.9% vs. 20.4% ± 1.1%), although the DeBakey Axial Flow VAD group was noted to have a collagen content of 14.3% ± 1.4% in comparison (*P *< 0.01). Finally, amounts of myocardial tumour necrosis factor‐α in the RV were significantly different (*P *< 0.001) between both the Novacor and DeBakey groups when compared with the no‐support cohort (3.0% ± 0.5%, 0.8% ± 0.1% and 35.4% ± 2.8%, respectively).

Barbone et al. (2001) also supported the notion of enhanced remodelling outcomes in the LV compared with the RV post‐LVAD implantation. They noted a further reduction in LV myocyte diameter compared with the RV (17% vs. 11%), with only changes in the LV being significant (*P* = 0.05). Additionally, LVAD implantation resulted in enhanced expression of sarcoplasmic/endoplasmic reticulum Ca^2+^‐ATPase 2a (SERCA2a) in the LV (*P *< 0.001) in comparison to the RV, increasing the levels of intracellular calcium.

On a molecular level, LVADs have also been associated with lower levels of matrix metalloproteinases, which has been linked to increased rates of HF, and increased expression of tissue inhibitors of metalloproteinases (Li et al., 2001). Additionally, LVADs have been correlated with the anti‐apoptosis protein Bcl‐XL, a protein thought to impact cardiac remodelling and myocardial hypertrophy (Bartling et al., 1999). The expression of nuclear factor kappa light chain has also been thought to impact levels of tumour necrosis factor‐α and matrix metalloproteinases, which can be altered with LVAD implantation (Grabellus et al., 2002). It is through mechanisms of both lower paracrine growth factor production and lower pulmonary arterial pressures and subsequent RV load that LVAD transplantation is thought to promote improved RV remodelling.

Notably, the extent of ventricular unloading might be influenced by the mechanism of the left ventricle assist system. For instance, in a further comparison between the DeBakey Axial Flow VAD, as mentioned above, and Novacor's LVAS, both devices were found to cause positive remodelling equally, reducing the mitral e‐wave to alpha‐wave ratio (−23.9% and −39.9%), tricuspid regurgitation velocity (−26.4% and −23.8%) and pulmonary valve acceleration time (28.5% and 38.5%), with no significant difference between these rates (Thohan et al., 2005). However, more favourable unloading was exhibited with the Novacor device, seen by reduced LV end‐diastolic dimension (−33.7% vs. −13.7%, *P* = 0.0.004), end‐diastolic volume (−41.2% vs. −23.5%, *P* = 0.015), end‐systolic volume (−57.6% vs. −25.6%, *P* = 0.001) and left atrial volume (−40.4% vs. −25.2%, *P* = 0.071).

Kato et al. (2011) confirm increased LV unloading in pulsatile devices compared with continuous flow devices, resulting in improved systolic and diastolic function. In their study, the pulsatile group had significantly improved LV ejection fractions compared to the continuous flow devices group (33.2% ± 12.6% vs. 17.6% ± 8.8%, *P *< 0.0001). Additionally, this study also reported decreased serum levels of brain natriuretic peptide postoperatively in the pulsatile device cohort (552.6 ± 340.6 vs. 965.4 ± 805.7 pg/mL, *P *< 0.01). However, it is essential to note that other literature has reported reduced survival rates with pulsatile devices (24%, in contrast to 58% at 2 years postoperatively, *P* = 0.008) (Slaughter et al., 2009). Furthermore, 46% of patients with an implanted continuous flow device were free from re‐intervention and disabling stroke after 2 years, compared with 11% in the pulsatile flow group (*P *< 0.001). LVAD‐associated infection was also higher with continuous LVADs (35% vs. 21%, *P* = 0.01).

Despite the promising outcomes described above, contraindications to VADs must be taken into account; this includes the severely multi‐morbid patient, including end‐stage renal failure, severe liver or lung disease, in addition to hypertrophic cardiomyopathy and significant ventricular septal defect, although some exceptions exist (Han et al., 2018). Additionally, patients who might not be suitable candidates for VAD implantation include those with co‐existing infections, active malignancy or bleeding (Frigerio, 2021).

### 2.2 Right ventricular assist devices

In a recent systematic review, right VADs (RVADs) were found to have a 30‐day survival between 46% and 100%, although studies with a sample size as small as 10 patients were included (Abdelshafy et al., 2022). Furthermore, between 23% and 100% of patients were fully weaned off RVADs successfully; this variability suggests the need for consideration of individual cases and indications accordingly. In comparison to LVADs, RVADs are designed at significantly lower afterloads to accommodate for pulmonary arterial pressures, and subsequently reduced hydraulic loads and power consumption rates. However, owing to the lower incidence of indications for RVADs, cardiac remodelling is not significantly detailed, including patients with biventricular failure or post‐LVAD RV failure (Karimov et al., 2016). The literature remains scarce regarding RVADs and their long‐term outcomes, and as the ageing population expands, the incidence of primary right‐sided heart failure and concomitant biventricular failure will continue to increase.

## 3 EXTRACORPOREAL MEMBRANE OXYGENATION

Veno‐arterial extracorporeal membrane oxygenation (VA‐ECMO) can be used to stabilize patients with haemodynamic compromise, with or without respiratory failure. Regarding cardiac pathology, ECMO can be implemented to treat CS, cardiac arrest and acute HF (Guglin et al., 2019; Tsuneyoshi & Rao, 2012). The ECMO machine acts as a life support, whereby patients are cannulated in major arteries and veins; a drainage cannula is introduced into a large vein (femoral vein or internal jugular vein) and a return cannula into a large artery (femoral artery or carotid artery or ascending aorta) (Pooboni & Gulla, 2021). VA‐ECMO can be used for heart and lung support, whereas venovenous ECMO is used for lung support only (Makdisi & Wang, 2015). The blood is pumped to an oxygenator, which acts as an artificial lung. The blood is pumped back to the patient, replacing the function of the heart. The specialist can adjust the machine such that the amount of support to the cardiopulmonary system can be tailored to the demands of the patient (White, 2016). It does not treat the underlying disease or injury, despite stabilizing the condition of the patient.

Theoretically, VA‐ECMO receives venous blood that is then returned to the arterial system via a main artery, such as the iliac artery. Hence, it reduces preload and increases aortic flow and end‐organ perfusion (Napp et al., 2016). In patients with CS, secondary to acute coronary syndrome, ischaemic heart failure, valvular disease and iatrogenic shock, VA‐ECMO was found to have 57% weaning success rate, although in‐hospital mortality has been reported to be as high as 69% (de Waha et al., 2016).

With regard to the role of VA‐ECMO in physiological change, the literature has revealed that the afterload was raised; this was attributed to retrograde flow towards the aortic valve generated by the arterial outflow cannula during the procedure (Dickstein, 2018; O'Keefe & Singh, 2024). It is also proposed that LV afterload is increased owing to the continuous flow of the VA‐ECMO and the resultant increase in MAP (Choi et al., 2019). Although VA‐ECMO confers afterload stress, it does not affect the Starling relationship, because the cardiopulmonary bypass mechanism and the reduction in pulmonary blood flow result in a predictable decrease in the LV preload. An increase in VA‐ECMO flow reduces LV stroke volume owing to the amount of native cardiopulmonary circulation, which decreases during cardiopulmonary bypass (Cevasco et al., 2019). An increased LV wall stress and distension induce a rise in intracardiac pressure. This high left ventricular end‐diastolic pressure causes ischaemia, hindering LV recovery (Werdan et al., 2014). With regard to the impact on coronary blood flow, no significant change has been found after initiation of ECMO (*P* = 0.49) (Kinsella et al., 1992).

Extracardiac pressures, such as PCWP, were likewise increased by the LV distension as the afterload increased (Dickstein, 2018). A simulation study investigating the intra‐aortic balloon pump (IABP) combined with VA‐ECMO showed a reduction in afterload when the IABP was used, although PCWP and left ventricular end‐diastolic volume were unchanged (Donker et al., 2019). However, it is difficult to extrapolate this finding until it is done on real patients.

It is increasingly recognized that VA‐ECMO promotes myocardial remodelling because it causes significant cardiac mechanical overload. This increases the strain exerted on the LV myocardium, which impedes the process of cardiac recovery. Hence, the potential consequence of myocardial remodelling is irreversible heart failure. Left ventricular dilatation can arise as a complication, in addition to increased filling pressure, exacerbating pulmonary oedema and hindering gas exchange (Donker et al., 2019). Moreover, LV contractility and recovery can become impaired owing to a vicious cycle of proximal aortic hypoxaemia and myocardial ischaemia with insufficient LV unloading and pulmonary oedema. The LV global longitudinal strain was found to be the highest during the acute phase of myocardial ischaemia, in contrast to the delayed phase (72.7% vs. 22.5%, respectively, *P *< 0.001), and with lower target blood flow parameters (−6.1% for 120% target blood flow, −8.8% during 50% blood flow, *P *< 0.001) (Ng et al., 2022). VA‐ECMO has also been linked to increased levels of circulating immature neutrophils and pro‐inflammatory cytokines, such as interleukin‐6, ‐8 and ‐10 and tumour necrosis factor‐alpha, indicating early immune changes predisposing patients to higher risks of infection (Frerou et al., 2021). Despite the benefits of ECMO, it is not a cure; instead, it is a process that includes several components, such as the appropriate selection of a suitable candidate, accurate diagnosis, prevention and management of complications, and provision of general critical care, to bridge cardiac patients to proper treatment. Further studies into the morphological and physiological changes following VA‐ECMO are required to understand this process better. Lastly, it is crucial to implement a multidisciplinary approach to ensure optimal outcomes.

## 4 IMPELLA (PERCUTANEOUS LEFT VENTRICULAR ASSIST DEVICE)

The Impella system (Abiomed, Danvers, MA, USA) is an intravascular microaxial blood pump used to provide temporary MCS (Zein et al., 2022). The Impella system is inserted percutaneously and is designed to be positioned across the aortic valve, with the inflow portion in the LV and the outflow portion in the aortic root (Glazier & Kaki, 2019). The two main indications for Impella are CS and high‐risk percutaneous cardiac intervention (PCI), with limited literature on Impella use in other clinical contexts. Newer versions of the device have also been designed for use in the right heart, lying across the pulmonary valve, with the inflow and outflow tracts residing in the right ventricle and pulmonary artery, respectively (Zein et al., 2022). The Impella system is a family of devices in different sizes of inlet and outlet cannulas capable of varying flow rate levels and circulatory support (from 2.5 to 5.5 L/min) (Zein et al., 2022). Implantation of the device is usually percutaneous, although surgical cutdown is necessary for larger Impella devices (Impella 5.0, 5.5, LD) owing to the larger cannula size (Papolos et al., 2022).

By facilitating continuous LV unloading, Impella subsequently reduces the LV end‐diastolic pressure (Burzotta et al., 2015). In a year‐long pilot study, Sjauw et al. (2008) reported a 30% reduction in LV afterload following Impella implantation. By decreasing the work done by the LV, the myocardial oxygen demand is reduced, hence limiting further myocardial ischaemia and apoptosis (Burzotta et al., 2015). A pilot trial of Impella patients having PCI in acute myocardial infarction (MI) reported improvements in end‐diastolic LV pressure and LV ejection fraction. The LV ejection fraction improved by nine percentage points after 72 h and 13 percentage points after 4 months; this is compared with two and three percentage points, respectively, for patients not supported by Impella (Sjauw et al., 2008). The sustained improvements in LV ejection fraction suggest that reductions in LV work offer myocardial protection and promote long‐term recovery.

The anterograde blood flow provided by Impella increases cardiac output and improves systemic perfusion, as seen in multiple studies. Seyfarth et al. (2008) reported that cardiac index increased from 1.71 to 2.2 L/min/m^2^ following Impella implantation in patients with CS. Analysis of the USpella registry demonstrated an improvement of 0.8 L/min/m^2^ in patients with acute MI complicated by CS (O'Neill et al., 2014). In addition, significant improvements in MAP are seen following Impella implantation. Data from the IMPRESS in Severe Shock trial showcased an increase in MAP of >20 mmHg (Ouweneel et al., 2017). Similar improvements in MAP are reported by O'Neill et al., with average MAP increasing from 62.7 to 94.4 mmHg after Impella insertion, and by Seyfarth et al., with average MAP increasing from 78 to 87 mmHg (O'Neill et al., 2014; Seyfarth et al., 2008). The improvement in MAP caused by Impella improves organ perfusion, evidenced by reduced lactate levels. Data from the EUROSHOCK‐Registry showed that Impella insertion was associated with reduction in plasma lactate from 5.8 mmol/L before insertion to 4.7 and 2.5 mmol/L at 24 and 48 h post‐insertion, respectively, in patients with CS (Lauten et al., 2013). Mean lactate levels decreased by over 5 mmol/L 8 h after Impella insertion in the IMPRESS in Severe Shock trial (Ouweneel et al., 2017). Moreover, improved organ perfusion with Impella reduces the risk of organ failure, as seen in the study by Seyfarth et al. (2008), mentioned above, wherein multi‐organ dysfunction at 30 days was fourfold lower relative to baseline.

The increase in systemic perfusion and LV unloading mediated by Impella increases coronary perfusion. Remmelink et al. (2007) report increased coronary pressure and hyperaemic flow velocity after Impella implantation during PCI. Two mechanisms are likely to mediate this improvement in coronary blood flow with Impella (Seyfarth et al., 2008). Firstly, Impella improves systolic blood pressure and aortic flow, increasing coronary perfusion. Secondly, LV unloading and subsequent reduction in end‐diastolic volume lead to reduced intramyocardial resistance (Burzotta et al., 2015; Remmelink et al., 2007). It is also important to point out that LV unloading reduces pulmonary pressures in patients requiring MCS, protecting the RV in acute LV failure. Data from the USpella registry show improvement in PCWP from 31.9 to 19.2 mmHg following Impella insertion (O'Neill et al., 2014). In the ISAR‐SHOCK trial, PCWP decreased from 22 mmHg preoperatively to 19 mmHg postoperatively (Seyfarth et al., 2008).

Complications associated with Impella insertion include bleeding and haematoma (Glazier & Kaki, 2019). There is also an increased risk of an embolic event, for which systemic anticoagulation is required (Papolos et al., 2022). Haemolysis must be monitored when patients are on Impella, because propulsion of blood through the device can cause haemolytic events.

The Impella devices are contraindicated in patients with severe peripheral arterial disease, owing to the risk of limb ischaemia following insertion (Wong & Sin, 2020). Obstructions of the aortic valve, such as mechanical aortic valves or aortic stenosis, can lead to difficulty achieving correct Impella position and are contraindications to Impella insertion. Impella use is also contraindicated in patients with LV thrombus, because clot fragments passing through the system can cause pump damage and, ultimately, device failure (Chera et al., 2018).

A newer device model, the Impella RP, has been designed for use on the right side of the heart. Data from the RECOVER RIGHT trial showed that Impella RP can improve haemodynamic parameters in patients with RV failure, including a reduction in central venous pressure from 19.2 ± 4 to 12.6 ± 1 mm Hg (*P* < 0.001), in addition to an increase in cardiac index from 1.8 ± 0.2 to 3.3 ± 0.23 L/min/m^2^ (*P* < 0.001) (Anderson et al., 2015).

### 4.1 Cardiogenic shock

Mechanical circulatory support by Impella in CS has been well described in the literature, whereby it has been shown to improve haemodynamic parameters, such as cardiac index, cardiac output and MAP, in patients with CS (Zein et al., 2022).

Two randomized controlled trials (RCTs) have compared the use of Impella with IABP in CS (Ouweneel et al., 2017; Seyfarth et al., 2008). Although improved haemodynamic indices were reported in patients supported by Impella, there was no difference in mortality. However, these studies were small and underpowered to detect effect sizes for their respective outcomes. A meta‐analysis including these trials and other observational studies confirmed that Impella did not improve mortality compared with IABP (Moustafa et al., 2022). Retrospective studies have confirmed the feasibility and safety of Impella use in CS (Lemaire et al., 2014). However, there are no clear guidelines for the optimal timing of intervention. For example, retrospective analysis of the USpella database comparing Impella implantation before or after PCI reported improved patient outcomes and revascularization in those receiving Impella before PCI complicated by CS (O'Neill et al., 2014). This study supports the role of Impella in this scenario and suggests that further research is necessary to maximize its benefits to patients with CS.

### 4.2 High‐risk PCI

Impella is also indicated for use in patients with severe coronary artery disease who require revascularization but are not suitable for surgery. These patients, whose only option is PCI, are termed high‐risk PCI. MCS support is used in this scenario to mitigate the risks of fatal ischaemia or dysrhythmia from the procedure. Impella is safe for implantation and use in this subset, with an in‐hospital mortality rate of 76% in patients with CS, and 8.3% in those who underwent high‐risk PCI (Burzotta et al., 2008; Pietrasik et al., 2023; Sjauw et al., 2008). However, further high‐quality research into morbidity and mortality in this group is vital. The PROTECT II RCT, comparing outcomes of patients treated with IABP or Impella 2.5 undergoing high‐risk PCI, showed improved haemodynamic indices with the Impella system but no difference in the 30‐day mortality or significant adverse events (which included stroke, further MI and renal failure). At the 90‐day interval, the Imeplla group had less major adverse events reported (40.6%), in contrast to the IABP group (49.3%), although this did not prove statistical significance (*P* = 0.066) (O'Neill et al., 2012), Impella has also been shown to be safe for use in ventricular tachycardia ablation (PERMIT1 study), providing better haemodynamic support than pharmacological support alone (Miller et al., 2013).

## 5 TANDEMHEART

TandemHeart is a centrifugal VAD inserted percutaneously. It comprises an inflow cannula in the left atrium, an outflow cannula in the abdominal aorta or femoral artery, and a pump (Kar et al., 2011). The inflow cannula is inserted into the left atrium via the femoral vein and transeptal puncture, and the outflow cannula is inserted by arterial access (Mandawat & Rao, 2017). By withdrawing blood from the left atrium and pumping it directly into the systemic circulation, TandemHeart facilitates LV unloading (Telukuntla & Estep, 2020). Reducing LV preload reduces LV workload and myocardial oxygen demand, improving myocardial recovery (Thiele et al., 2005).

Although not without its complications, TandemHeart is a safe and feasible option in high‐risk PCI. A retrospective evaluation of outcomes of patients undergoing high‐risk PCI with TandemHeart support showed procedure success rates of 77% (Neupane et al., 2020). Other less common uses of TandemHeart include combination with VA‐ECMO for acute severe mitral regurgitation and bridge therapy for transplantation (Bruckner et al., 2008; DiVita et al., 2020).

The MCS provided by TandemHeart improves cardiac output and supports MAP, increasing systemic perfusion. In a randomized control study in patients with acute MI complicated by CS, the cardiac index improved by 0.5L/min/m^2^, and MAP increased by 7.5 mmHg following insertion of TandemHeart, compared with IABP (Burkhoff et al., 2006). A second RCT supported these findings, reporting that the cardiac index improved by 0.5 L/min/m^2^, and the cardiac power index improved by 0.15 W (Thiele et al., 2005). A retrospective analysis of patients with ischaemic and non‐ischaemic cardiomyopathy showed significant improvements in MAP after TandemHeart insertion. In ischaemic cardiomyopathy, MAP increased from 40 to 82 mmHg, and in non‐ischaemic cardiomyopathy, MAP increased from 55 to 80.5 mmHg, which was associated with significant improvements in serum lactate in both groups (Kar et al., 2011).

Left ventricular offloading caused by TandemHeart also reduces pulmonary pressures, evident in two RCTs that reported improvements in PCWP of 4–11 mmHg following TandemHeart insertion for CS (Burkhoff et al., 2006; Thiele et al., 2005). Similar to Impella, the two main indications for TandemHeart are CS and high‐risk PCI. Studies have assessed the utility of TandemHeart in CS secondary to acute MI. Use of TandemHeart results in improved haemodynamic and metabolic values compared with traditional MCS (e.g., IABP), which does not translate to reduced mortality. An RCT by Thiele et al. (2005) comparing TandemHeart with IABP in patients with acute MI causing CS found that TandemHeart use was associated with improved haemodynamic indices but no improvement in mortality. A further RCT investigated TandemHeart and IABP in patients with acute MI and CS. Again, although TandemHeart support was associated with better haemodynamic parameters, it resulted in similar mortality; cardiac indices in patients with non‐ischaemic and ischaemic cardiomyopathy suggest a role for TandemHeart in non‐ischaemic causes of CS (Burkhoff et al., 2006; Kar et al., 2011).

Potential complications of TandemHeart include bleeding and haematoma from insertion. Given the placement in systemic arteries, patients are also at risk of limb ischaemia (Wong & Sin, 2020). With that mentioned, using peripheral angiography before arterial cannulation has been suggested to reduce rates of limb ischaemia related to TandemHeart use (3.4% vs. 33%) (Kar et al., 2011). However, the device is contraindicated in severe peripheral arterial disease. Moreover, there are notable risks associated with a trans‐septal puncture, including cardiac tamponade and shunt creation (Wong & Sin, 2020). Lastly, intra‐atrial thrombus can impair the blood intake by the device from the left atrium, constituting a caution for its use (Wong & Sin, 2020).

## 6 OTHER CONSIDERATIONS

### 6.1 Haemodynamic response during exercise following MCS implantation

Reduced exercise tolerance is a hallmark feature of heart failure. Cardiac rehabilitation has been an integral part of the recovery of ischaemic heart disease and heart failure patients, promoting improved independence in performing activities of daily living and overall quality of life through exercise training (Toda et al., 2004). Although survival might improve with implantation of MCS devices, it is unclear whether improved functional capacity also ensues (Huang et al., 2022). Rogers et al. (2010) reported that following continuous flow LVAD insertion, 80% of patients bridged to transplant, and 82% of those for destination therapy exhibited improvement from a New York Heart Association function class IV to I or II after 6 months. At 6 months, other scores, including the Minnesota living with heart failure questionnaire, showed 52% improvement, whilst the Kansas City cardiomyopathy questionnaire showed improvement by 170%. A 2021 study that investigated patients with invasive cardiopulmonary exercise testing, using Swan–Ganz and conductance catheters, revealed that LVAD patients exhibited RV dysfunction during exercise, with limited increase in cardiac output and stroke volume, when compared with healthy candidates (Tran et al., 2021). Although post‐LVAD exercise tolerance will vary based on individual patient factors, including baseline mobility, age and other comorbidities, alongside device‐specific variations, such as blood flow velocity, levels of maximal oxygen uptake during cardiopulmonary exercise testing have shown no significant improvement. Leibner et al. (2013) reported no statistically significant change in mean maximal oxygen uptake prior to LVAD implantation (11.2 ± 3.0 mL/kg/min) and 1 year postimplantation (10.7 ± 2.6 mL/kg/min). Despite improved subjective exercise tolerance measures post‐LVAD, haemodynamic studies have evidenced limitations in augmenting blood flow during exercise. From rest to exercise points, LVAD flow was shown to increase by only 1.0−1.5 L/min, although total cardiac output increased by 4−5 L/min, suggesting that it is the native ventricle that is generating this discrepancy, as opposed to the MCS device (Huang et al., 2022). Other haemodynamic studies have suggested a significant rise in pulsatility in blood pressure waveforms without a dicrotic notch during exercise (Sailer et al., 2021). In addition, LVADs are thought to have reduced preload sensitivity, in contrast to the native ventricle, indicating that these MCS devices provide insufficient LV unloading during exercise (Fukamachi et al., 2013).

### 6.2 Optimal timing of MCS implantation

Patient selection and timing of MCS implantation are exceptionally important when patients begin to exhibit signs of clinical deterioration, worsening renal function, cardiac cachexia and deteriorating RV function (Maly, 2024). The optimal timing for the insertion of MCS devices remains controversial. The European Society of Cardiology guidelines advise temporary MCS in non‐acute, high‐risk PCI, including complex chronic total occlusions and single‐remaining patent coronary artery (Windecker et al., 2014). Left ventricular unloading in post‐ST segment MI patients with Impella followed by delayed reperfusion with PCI evidenced reduced reperfusion injury (Kapur et al., 2019). Further evidence‐based guidance is vital to improve LV unloading and remodelling outcomes.

## 7 CONCLUSION

Although HF represents a dynamic clinical challenge, the evolving nature of MCS offers a variety of means to overcome this through different devices. These facilitate the unloading of the ventricular failure to varying extents, which aids the ventricular remodelling process. The choice of MCS strategy should be based on individual patient factors, including indication, to optimize clinical outcomes. Limitations of the script were related to the small number of large, high‐quality RCTs, and therefore encompassed several narrative and retrospective data reports as a result, in addition to its nature as a literature review, rather than a systematic review, and did not include all literature available. Further studies are required to improve understanding of the LV remodelling process and subsequent unloading in different MCS devices.

## AUTHOR CONTRIBUTIONS

Conception or design of the work: Wael I. Awad, Mohamad Bashir, Ian M. Williams, Damian M. Bailey and Matti Jubouri. Acquisition, analysis or interpretation of data for the work: Fatima Kayali, Owais Tahhan, Guglielmo Vecchio, Matti Jubouri and Judi M. Noubani. Drafting of the work or revising it critically for important intellectual content: All authors. All authors read and approved the final version of this manuscript and hence qualify for authorship, and all those who qualify for authorship are listed. All authors agree to be accountable for all aspects of the work in ensuring that questions related to the accuracy or integrity of any part of the work are appropriately investigated and resolved.

## CONFLICT OF INTEREST

The authors declare no conflicts of interest.

## Data Availability

The evidence to support this article is publicly available in electronic databases, such as PubMed, Google Scholar, Ovid, Scopus and Embase.
